# Stage-Related Changes in TGF-β Isoforms in PBMC Culture Supernatants in Endometriosis: A Prospective Case–Control Study

**DOI:** 10.3390/ijms27093898

**Published:** 2026-04-27

**Authors:** Marcin Sadlocha, Jakub L. Toczek, Jakub Staniczek, Zenon Czuba, Rafal Stojko

**Affiliations:** 1 Department of Gynecology, Obstetrics and Gynecologic Oncology, Faculty of Health Sciences, Medical University of Silesia in Katowice, 40-055 Katowice, Polandjstaniczek@sum.edu.pl (J.S.);; 2 Department of Microbiology and Immunology, Faculty of Medical Sciences in Zabrze, Medical University of Silesia in Katowice, 40-055 Katowice, Poland; zczuba@sum.edu.pl

**Keywords:** endometriosis, TGF-β, TGF-β1, TGF-β2, TGF-β3, cytokines, PBMC, biomarkers, peritoneal microenvironment, in vitro

## Abstract

Endometriosis is a chronic inflammatory disease in which transforming growth factor-beta (TGF-β) has been implicated in immune dysregulation, extracellular matrix remodeling, and fibrosis. Data on baseline secretion of TGF-β isoforms by systemic immune cells remain limited. This pilot study quantified unstimulated secretion of TGF-β1, TGF-β2, and TGF-β3 by peripheral blood mononuclear cell (PBMC) cultures from women with and without endometriosis and explored stage-related patterns. In this prospective case–control study, PBMCs from 50 women with surgically confirmed endometriosis and 30 controls were cultured for 24 h without exogenous stimulation. Supernatant concentrations were measured using a multiplex bead-based immunoassay (Bio-Plex, Bio-Rad) and expressed as pg/mL; between-group and stage-related differences were assessed using non-parametric tests. Median 24 h secretion was similar between groups (TGF-β1: 103,816 vs. 114,700 pg/mL, *p* = 0.25; TGF-β2: 3735 vs. 3732 pg/mL, *p* = 0.32; TGF-β3: 3280 vs. 3284 pg/mL, *p* = 0.70). Within the endometriosis cohort, TGF-β2 was significantly higher in moderate/advanced disease (rASRM stages III–IV) than in minimal/mild disease (stages I–II), whereas TGF-β1 and TGF-β3 did not reach statistical significance for a stage-dependent pattern in this pilot cohort (*p* = 0.42 and *p* = 0.41, respectively; Kruskal–Wallis), and a type II error cannot be excluded given the small sample size per rASRM (revised American Society of Reproductive Medicine)stage (n = 11–14). These findings suggest that TGF-β dysregulation is compartmentalized to the peritoneal environment rather than systemically imprinted in circulating immune cells. The stage-dependent elevation of TGF-β2 supports its role in progressive fibrogenesis and as a candidate severity biomarker, warranting confirmation in larger, stimulus-augmented studies.

## 1. Introduction

Endometriosis is a chronic, estrogen-dependent inflammatory disease defined by the ectopic implantation of functional endometrial glands and stroma outside the uterine cavity, predominantly within the pelvic peritoneum, ovaries, and rectovaginal septum [1,2,3]. It affects approximately 10–19% of women of reproductive age worldwide and constitutes one of the most common causes of chronic pelvic pain, dysmenorrhea, dyspareunia, and female infertility. Endometriosis is identified in 30–50% of women undergoing assisted reproductive technology (ART), underscoring its profound impact on reproductive outcomes. Despite considerable research investment, the precise molecular mechanisms driving disease initiation, progression, and stage-dependent behavior remain incompletely understood, and the average diagnostic delay from symptom onset to confirmed diagnosis remains 7–10 years [1,2,3].

The most widely accepted pathogenic framework is the retrograde menstruation hypothesis, first formalized by Sampson in 1927, which proposes that viable endometrial fragments shed during menstruation travel retrogradely through the fallopian tubes and implant on peritoneal surfaces [4,5]. Supporting this model, recent single-cell DNA sequencing studies have demonstrated identical somatic mutations in eutopic and ectopic endometrial epithelial cells in hundreds of patients [6]. However, retrograde flow occurs physiologically in approximately 76–90% of women with patent tubes, yet only a minority develop endometriosis—implicating fundamental peritoneal immune surveillance failure as a necessary permissive condition [7]. Complementary theories, including coelomic metaplasia, Müllerian duct remnant activation, and bone-marrow-derived stem cell mislocalization, account for extraperitoneal manifestations and disease in patients with structural anomalies [4,5,6,7].

At the molecular level, the pathological microenvironment of endometriosis is orchestrated by a dysregulated cytokine network. Aberrant secretion of pro-inflammatory mediators—including interleukins (IL-1β, IL-6, IL-8), tumor necrosis factor-alpha (TNF-α), vascular endothelial growth factor (VEGF), and members of the transforming growth factor-beta (TGF-β) superfamily—drives ectopic cell adhesion, epithelial-to-mesenchymal transition (EMT), neoangiogenesis, extracellular matrix (ECM) remodeling, and fibrogenesis [2,8,9,10]. Among these mediators, TGF-β occupies a central position as both an immunosuppressive signal and a potent driver of fibrosis, invasion, and angiogenesis—processes that collectively define the invasive phenotype of endometriotic lesions [8,10].

### 1.1. The TGF-β Superfamily: Molecular Architecture and Signaling

The TGF-β superfamily comprises more than 30 structurally related ligands, including the three human TGF-β isoforms (TGF-β1, TGF-β2, TGF-β3), bone morphogenetic proteins (BMPs), activins, inhibins, and growth differentiation factors (GDFs) [8,10]. All three isoforms are synthesized as large precursors cleaved to yield a mature dimeric ligand non-covalently associated with a latency-associated peptide (LAP), forming the small latent complex (SLC). Extracellular activation occurs upon LAP dissociation triggered by matrix metalloproteinases (MMPs), thrombospondin-1 (TSP-1), reactive oxygen species (ROS), and integrin-mediated mechanical forces.

Active TGF-β signals via a heterotetrameric receptor complex of type I (TGFBR1/ALK5) and type II (TGFBR2) serine/threonine kinase receptors. Upon ligand-induced TGFBR2-mediated transphosphorylation of TGFBR1, canonical signaling proceeds via SMAD2/3 phosphorylation, co-SMAD4 complex formation, and nuclear translocation to drive transcriptional programs governing ECM deposition, growth arrest, and immune suppression [11,12,13,14,15]. Non-canonical pathways—including MAPK/ERK, PI3K/AKT, RhoA/ROCK, focal adhesion kinase (FAK), and Wnt/β-catenin cascades—are equally critical in endometriosis, contributing to EMT, cytoskeletal reorganization, and invasive cell migration. Notably, a non-canonical KLF11-mediated TGF-β pathway drives endometriotic progression by epigenetically repressing CYP3A4 via HDAC recruitment [15]. Furthermore, the TGF-β1–SMAD3–integrin-linked kinase (ILK) signaling axis, modulated by miR-21, has been identified as a key molecular determinant of EMT in eutopic and ectopic endometrium [16].

### 1.2. TGF-β1: Immunosuppression, EMT, and Ectopic Cell Survival

TGF-β1 is the most abundantly expressed and most extensively characterized TGF-β isoform in endometriosis. Significantly elevated TGF-β1 concentrations have been documented in the peritoneal fluid, serum, ectopic endometrium, and peritoneal tissue of affected women compared to controls [9,10,11,17]. At the cellular level, TGF-β1 facilitates adhesion of endometrial epithelial cells (EECs) to peritoneal mesothelium through integrin modulation and enhances transmesothelial invasion through c-fms upregulation and MMP-mediated ECM degradation. TGF-β1 overexpression significantly increases endometrial stromal cell (ESC) proliferation by upregulating PCNA and Cyclin D1 expression via MAPK/ERK-dependent pathways, while conferring anti-apoptotic resistance through c-Myc and Bcl-2 family upregulation [10,16,17,18].

From an immunological standpoint, TGF-β1 is a master immunosuppressive cytokine within the peritoneal microenvironment. It promotes Foxp3+ regulatory T cell (Treg) induction, suppresses NK cell cytotoxicity, modulates the Treg/Th17 axis in favor of immune tolerance, and polarizes peritoneal macrophages toward an M2 pro-fibrotic phenotype—collectively shielding ectopic implants from immune-mediated clearance [9,17,19]. Despite this mechanistic centrality, studies on circulating TGF-β1 levels have yielded inconsistent results: whereas some authors report significantly elevated concentrations in peritoneal fluid and serum, others have not detected statistically significant differences in PBMC-derived TGF-β1 output [9,11,17]. These discrepancies likely reflect methodological heterogeneity, stage distribution, menstrual cycle phase at sampling, and the intrinsic limitations of isoform-nonspecific assays.

### 1.3. TGF-β2: A Stage-Dependent Driver of Fibrosis and Ovarian Pathology

TGF-β2 appears to be an important mediator of myofibroblast differentiation, fibroblast proliferation, migration, and pathological ECM deposition through canonical SMAD2/3 signaling and non-canonical Wnt/β-catenin, FAK, and RhoA/ROCK pathways [8,12]. It plays a particularly critical role in ovarian endometrioma (OMA) formation, where ectopic ESCs synthesize and release TGF-β2 that accumulates in surrounding ovarian parenchyma, inducing progressive cortical fibrosis and fibrous adnexal adhesions [12,20]. Therapeutic targeting of the TGFBR2 subunit via neutralizing monoclonal antibodies, gene-based vaccines, or small-molecule kinase inhibitors has shown capacity to suppress fibrogenesis and ESC invasion in preclinical models. From a biomarker perspective, TGF-β2 is a plausible candidate for stage-related analyses because of its documented links to fibrosis and disease progression in endometriosis [1,8].

### 1.4. TGF-β3: Fibrogenesis, Th17 Activation, and Reproductive Failure

TGF-β3 contributes to the fibrogenic milieu of endometriosis through overlapping yet mechanistically distinct mechanisms. Unlike TGF-β1, which in combination with IL-2 favors Treg induction, TGF-β3 in concert with pro-inflammatory signals promotes Th17 lymphocyte differentiation, linking it to the amplification of local inflammatory loops [1,8]. Elevated TGF-β3 expression may amplify retrograde menstruation-associated inflammation and extend the ectopic survival of endometrial fragments. Additionally, abnormal TGF-β3 concentrations in the uterine microenvironment have been associated with repeated implantation failure (RIF) and chronic endometritis, implicating this isoform in the reproductive sequelae of endometriosis [1,8].

### 1.5. Peripheral Blood Mononuclear Cells as an Immunological Model System

Peripheral blood mononuclear cells (PBMCs)—comprising T lymphocytes (CD4+/CD8+), B lymphocytes, NK cells, and monocytes—represent an accessible and immunologically informative compartment for studying the systemic immune dysregulation of endometriosis [20,21,22]. In vitro PBMC cultures and their conditioned supernatants offer a standardized model of peripheral immune secretory phenotype, avoiding the confounding effects of matrix-bound cytokines present in serum. The methodological validity of this approach in endometriosis research was established by Sadocha et al., who demonstrated significantly elevated MIP-1α and MIP-1β concentrations (*p* = 0.00001 and *p* = 0.000026, respectively) in PHA-stimulated lymphocyte culture supernatants from endometriosis patients compared to controls [23]. The Bio-Plex multiplex immunoassay platform (Bio-Rad Laboratories, Hercules, CA, USA), utilizing xMAP suspension array technology, enables simultaneous quantification of multiple cytokine isoforms from single supernatant aliquots with sensitivity and reproducibility comparable to conventional ELISA [20,21,22,24].

Systematic stage-stratified quantification of all three TGF-β isoforms from PBMC supernatants using validated multiplex methodology has not previously been reported, constituting the specific evidence gap addressed by the present study [21,25].

### 1.6. Diagnostic Landscape and the Unmet Need for Blood-Based Biomarkers

The definitive diagnosis of endometriosis currently requires laparoscopic visualization with histopathological confirmation—an invasive approach with a protracted diagnostic pathway averaging 7–10 years [1,2,3]. Non-invasive imaging modalities, including transvaginal ultrasound (TVS) and magnetic resonance imaging (MRI), have substantially improved preoperative mapping of deep infiltrating endometriosis (DIE) and ovarian endometriomas but retain limited sensitivity for peritoneal implants and early-stage disease [3,23]. The serum biomarker CA-125 exhibits insufficient sensitivity and specificity for reliable diagnostic application across all rASRM stages. TGF-β isoforms—particularly given their mechanistic centrality in endometriosis pathogenesis, their expression in accessible peripheral blood compartments, and the emerging stage-dependent behavior of TGF-β2—represent a biologically compelling candidate class for minimally invasive biomarker development [2,8,10].

### 1.7. Aims of the Study

The primary objective of this prospective case–control study was to quantify TGF-β1, TGF-β2, and TGF-β3 concentrations in PBMC culture supernatants from women with laparoscopically and histopathologically confirmed endometriosis (rASRM stages I–IV) and age-matched controls. Secondary objectives were (i) evaluation of isoform-specific concentration differences between study and control groups; (ii) characterization of stage-dependent dynamics across the rASRM spectrum; (iii) analysis of correlations between TGF-β isoform concentrations and clinical variables including age, number of pregnancies, and vaginal deliveries; and (iv) assessment of inter-isoform correlations within and between groups to delineate potential synergistic or dissociative functional relationships. The hypothesis that TGF-β isoforms exhibit distinct, stage-related secretion patterns in PBMC supernatants could inform the development of a non-invasive peripheral immune profiling strategy and for the rational prioritization of isoform-targeted therapies in endometriosis [8,10,23].

## 2. Results

### 2.1. Participant Characteristics

Eighty women were enrolled in this prospective study: 50 with laparoscopically and histopathologically confirmed endometriosis and 30 disease-free controls. The mean age was 34.2 ± 8.2 years (median 35 years) in the endometriosis group and 37.5 ± 11.7 years (median 35.5 years) in the control group, with no statistically significant difference between groups (*p* = 0.45). The clinical and demographic characteristics of the study participants are presented in Table 1.

Nulligravidity was significantly more frequent in the endometriosis group compared to controls (60.0% vs. 36.7%; *p* = 0.04), consistent with the known association between endometriosis and subfertility. Within the endometriosis cohort, disease stage distribution according to the revised American Society for Reproductive Medicine (rASRM) classification was as follows: stage I (minimal)—12 patients (24%); stage II (mild)—14 patients (28%); stage III (moderate)—13 patients (26%); stage IV (severe)—11 patients (22%).

Controls were women who had undergone diagnostic or operative laparoscopy for benign indications unrelated to endometriosis (e.g., tubal ligation, diagnostic evaluation of pelvic pain) and in whom no endometriotic lesions were identified at laparoscopic inspection, thereby fulfilling the criterion of laparoscopically confirmed absence of endometriosis. This recruitment strategy is consistent with established methodological standards for endometriosis case–control studies, in which a negative laparoscopy represents the most accurate non-histological means of excluding the disease [26]. Accordingly, the possibility of undetected subclinical endometriosis in controls, while theoretically non-zero, is minimized by the use of direct surgical visualization.

### 2.2. TGF-β Isoform Concentrations: Endometriosis vs. Controls

Median 24 h in vitro TGF-β secretion by unstimulated PBMCs was comparable between the endometriosis and control groups for all three isoforms (Table 2). For TGF-β1, median concentrations were 103,816 pg/mL (IQR 90,955–125,630) in the endometriosis group and 114,700 pg/mL (IQR 96,800–121,671) in controls (*p* = 0.25). TGF-β2 concentrations were 3735 pg/mL (IQR 3142–4204) and 3732 pg/mL (IQR 3362–4179), respectively (*p* = 0.32). For TGF-β3, median values were 3280 pg/mL (IQR 3027–3463) in the endometriosis group and 3284 pg/mL (IQR 3050–3477) in controls (*p* = 0.70). None of the between-group differences reached statistical significance at α = 0.05, indicating that basal TGF-β secretory capacity of circulating immune cells does not differ globally between women with and without endometriosis.

The approximately 30-fold higher median concentration of TGF-β1 relative to TGF-β2 and TGF-β3 in both groups is consistent with established isoform-specific biology. TGF-β1 is the dominant immunoregulatory isoform expressed by haematopoietic cells—particularly CD4+ regulatory T cells, monocytes/macrophages, and NK cells—where its constitutive secretion serves as a major homeostatic immunosuppressive signal [27,28]. In contrast, TGF-β2 and TGF-β3 are more prominently expressed in non-immune mesenchymal compartments (including neural, chondrocytic, and epithelial cells) and are predominantly involved in fibrogenic and morphogenetic processes rather than systemic immune regulation [29,30]. Furthermore, TGF-β2 exhibits substantially lower affinity for the canonical TβRII receptor and may require accessory coreceptors such as betaglycan for efficient signaling complex assembly, potentially limiting its basal secretory output in isolated PBMC cultures lacking mesenchymal co-stimulatory cues. Recent evidence from lung and liver fibrosis models demonstrates that TGFB2 and TGFB3 expression is selectively elevated in fibrotic tissue in situ, without a corresponding increase in circulating immune cell secretion [31]. Collectively, the observed concentration hierarchy—TGF-β1 ≫ TGF-β2 ≈ TGF-β3—thus reflects intrinsic PBMC biology rather than a disease-specific phenomenon, and supports the interpretation that TGF-β2 and TGF-β3 elevation in endometriosis is primarily a local, tissue-compartmentalized event driven by peritoneal and lesion-specific fibrogenic stimuli.

**Figure 1:**
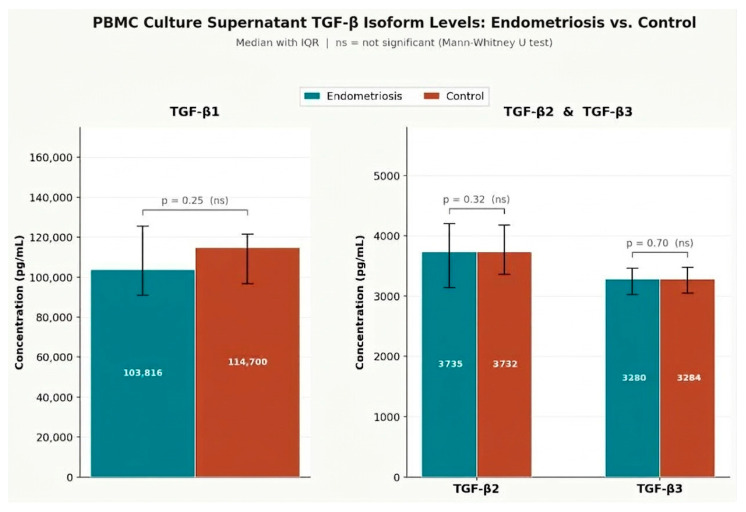
Supernatants levels of TGF-β1, TGF-β2, and TGF-β3 in endometriosis patients and healthy controls. Bars represent medians; error bars indicate IQR. TGF-β1 is shown on a separate scale. Groups were compared using the Mann–Whitney U test. No significant differences were found (*p* = 0.25, *p* = 0.32, *p* = 0.70, respectively). ns—not significant.

Figure 1 illustrates the distribution of TGF-β isoform concentrations across study groups, showing virtually overlapping medians and interquartile ranges for all three isoforms.

### 2.3. Stage-Related Analysis of TGF-β Isoforms

**Figure 2:**
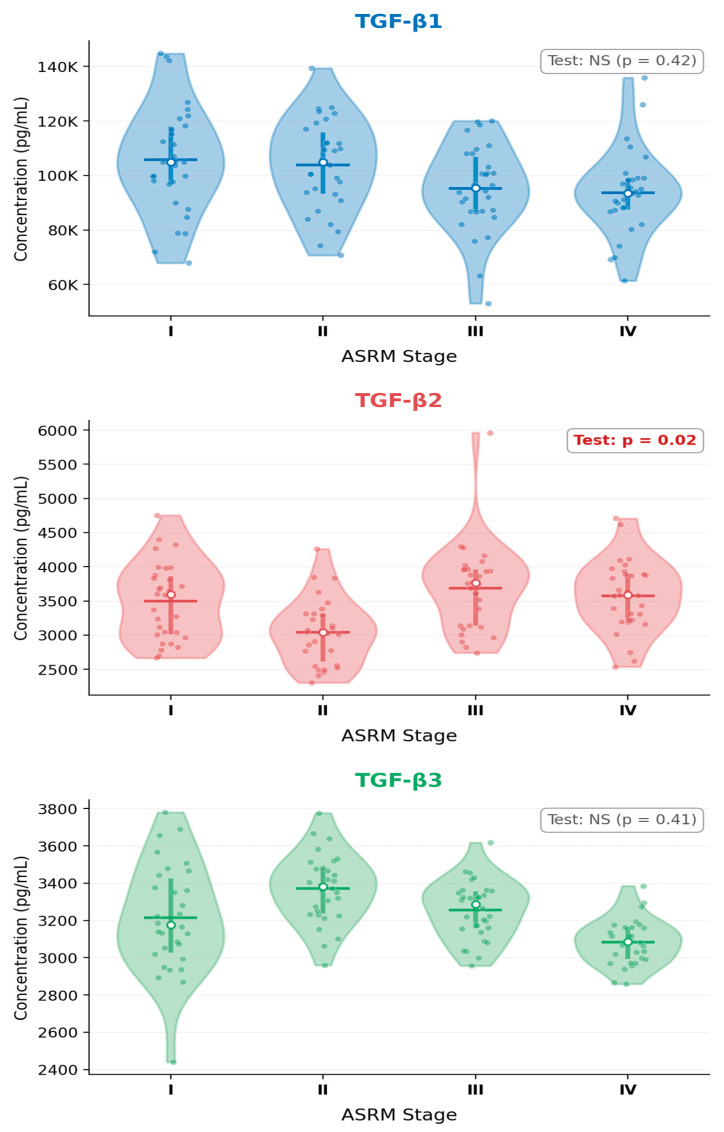
Supernatants concentrations of TGF-β1, TGF-β2, and TGF-β3 by revised ASRM endometriosis stage (I–IV). Violin plots display the data distribution, interquartile range (thick line), median (white circle), and individual values. TGF-β2 levels differed significantly across stages (*p* = 0.02), whereas TGF-β1 (*p* = 0.42) and TGF-β3 (*p* = 0.41) did not. Kruskal–Wallis test; NS—not significant.

The distribution of TGF-β isoform concentrations across rASRM stages is shown in Figure 2. Within the endometriosis cohort, Kruskal–Wallis analysis across rASRM stages revealed a statistically significant difference in TGF-β2 concentrations (*p* < 0.05), with post hoc pairwise comparisons demonstrating significantly higher TGF-β2 levels in women with moderate-to-advanced disease (rASRM stages III–IV) compared with those with minimal-to-mild disease (stages I–II). By contrast, TGF-β1 and TGF-β3 did not reach statistical significance for a stage-dependent pattern in this pilot cohort (Table 3).

### 2.4. Correlations with Clinical Variables

Spearman’s rank correlation analysis revealed no statistically significant associations between any TGF-β isoform concentration and age (all ρ < 0.15, *p* > 0.10), number of pregnancies (all ρ < 0.18, *p* > 0.10), or number of vaginal deliveries (all ρ < 0.16, *p* > 0.10) in either the endometriosis or control group. Inter-isoform correlation analysis within the endometriosis cohort demonstrated moderate positive correlations between TGF-β1 and TGF-β3 (ρ = 0.62, *p* < 0.001) and between TGF-β2 and TGF-β3 (ρ = 0.44, *p* < 0.01), whereas the TGF-β1–TGF-β2 correlation was weaker (ρ = 0.31, *p* = 0.03). In the control group, all inter-isoform correlations were strong and comparable (ρ range 0.71–0.78, all *p* < 0.001), suggesting that the dissociation of TGF-β2 secretion from the co-regulated TGF-β1/TGF-β3 cluster may be a disease-specific phenomenon reflecting the differential involvement of TGF-β2 in advanced-stage fibrotic pathology.

## 3. Discussion

The principal finding of this prospective case–control study is that unstimulated 24 h PBMC cultures from women with laparoscopically confirmed endometriosis do not exhibit globally elevated TGF-β secretion compared with disease-free controls. Median concentrations of TGF-β1, TGF-β2, and TGF-β3 were virtually identical between groups (all *p* > 0.25), indicating that circulating immune cells, under basal conditions, do not carry an intrinsically reprogrammed TGF-β secretory phenotype detectable at the whole-PBMC level. This negative finding is of biological significance, as it contrasts with the consistently reported elevation of TGF-β1 in peritoneal fluid, ectopic endometrium, and peritoneal mesothelium of affected women [9,11,17], and strongly supports a model in which TGF-β dysregulation in endometriosis is predominantly compartmentalized to the peritoneal and lesion microenvironment rather than systemically imprinted in the peripheral immune compartment. The potential confounding effect of menstrual cycle phase on PBMC-derived TGF-β secretion is partially mitigated by the preferential scheduling of blood collection during the early-to-mid follicular phase (cycle days 5–12), the phase in which endometrial TGF-β2 expression is at its nadir, thereby reducing the likelihood that cyclical TGF-β2 peaks artefactually contributed to the stage-dependent signal observed in this study.

A future study incorporating parallel peritoneal fluid collection from the same patients would constitute the most direct test of the compartmentalization hypothesis and should be regarded as a priority next step for this line of research.

The concept of compartmentalized cytokine dysregulation aligns with accumulating evidence that peritoneal mesothelial cells, ectopic endometrial stromal cells, M2-polarized macrophages, and regulatory T cells within the peritoneal cavity constitute the major local sources of TGF-β1 overproduction [9,11,21]. Young et al. demonstrated that peritoneal mesothelium adjacent to endometriotic lesions expresses significantly higher TGF-β1 mRNA than distant sites, indicating a paracrine gradient confined to lesion proximity [11]. Similarly, the systematic review by Xu et al. confirmed that TGF-β superfamily members are preferentially elevated in peritoneal fluid relative to matched serum samples [9]. Our finding that PBMCs cultured in isolation from peritoneal signals do not recapitulate this elevation is therefore biologically coherent: it suggests that the peripheral immune system requires local peritoneal cues—including direct cell contact with ectopic endometrial tissue, peritoneal fluid-borne inflammatory mediators, and hypoxic microenvironmental signals—to upregulate TGF-β secretion. This has important implications for biomarker development, as it implies that assay systems that physically isolate PBMCs from peritoneal stimuli may underestimate the true immunological deviation of endometriosis unless stimulation protocols are employed.

Despite the absence of overall group differences, a noteworthy secondary finding was the stage-dependent increase in TGF-β2 concentrations within the endometriosis cohort. Women with moderate-to-advanced disease (rASRM stages III–IV) showed significantly higher in vitro TGF-β2 secretion than those with minimal-to-mild disease (stages I–II), whereas TGF-β1 and TGF-β3 did not exhibit a comparable stage-related gradient. This isoform-specific behavior is consistent with the established role of TGF-β2 as the principal driver of myofibroblast differentiation, fibroblast proliferation, and pathological extracellular matrix deposition in ovarian endometrioma formation via the canonical SMAD2/3 signaling axis [12,20]. Shi et al. specifically demonstrated that TGF-β1 derived from endometriomas promotes progressive cortical fibrosis in surrounding ovarian tissue through Smad2/3 phosphorylation [20], and the TGF-β2/SMAD axis has been shown to directly promote ovarian fibrosis and fibrous adnexal adhesion formation [12].

The detection of a stage-related TGF-β2 signal even in unstimulated peripheral cultures raises the possibility that advanced disease induces a degree of systemic immune priming that is isoform-selective. This may reflect chronic exposure of circulating monocytes and lymphocytes to recirculating peritoneal mediators as disease burden increases, an effect known as peripheral immune ‘spillover’ from a heavily inflamed peritoneal niche. The progressive accumulation of fibrous deposits, adhesions, and endometrioma-derived TGF-β2 in moderate-to-advanced stages may raise systemic TGF-β2 concentrations to levels sufficient to prime PBMC secretory phenotype even in basal culture conditions. This is further supported by the relative dissociation of TGF-β2 from the tightly co-regulated TGF-β1/TGF-β3 cluster observed in the inter-isoform correlation analysis: while TGF-β1 and TGF-β3 remained strongly correlated in both groups (ρ > 0.60 in endometriosis, ρ > 0.71 in controls), the TGF-β1–TGF-β2 correlation was significantly weaker in the endometriosis group (ρ = 0.31), suggesting that TGF-β2 regulation is decoupled from the remaining isoforms in the disease state—a pattern that may reflect its selective induction by fibrosis-specific stimuli such as hypoxia, reactive oxygen species accumulation, and mechanical ECM strain within advanced lesions [8,12,13].

These findings have direct translational relevance. The stage-dependent behavior of TGF-β2 in PBMC supernatants suggests a potential role not as a primary diagnostic marker for endometriosis detection, but as a candidate biomarker of disease severity and fibrotic burden. This is particularly pertinent in the context of ovarian reserve monitoring in patients with endometrioma, where progressive fibrotic loss of primordial follicles is a major clinical concern. Longitudinal tracking of PBMC-derived TGF-β2 concentrations before and after surgical excision or anti-fibrotic therapy may be worth evaluating as a potential minimally invasive indicator of lesion activity in future longitudinal studies.

The lack of a stage-dependent pattern for TGF-β1 in our PBMC supernatants deserves particular comment, given that TGF-β1 is the most extensively characterized isoform in endometriosis pathophysiology. Elevated TGF-β1 has been consistently documented in peritoneal fluid, serum, and ectopic tissue [9,11,17], and TGF-β1 signaling is mechanistically central to immune evasion through Foxp3+ Treg induction, NK cell suppression, and macrophage M2 polarization [10,19,25]. However, our protocol employed unstimulated cultures without exogenous mitogenic or antigenic stimulation, which captures only the constitutive (basal) secretory capacity of PBMCs rather than their inducible potential.

Earlier work from our group demonstrated that PHA-stimulated lymphocyte cultures unmask significant group differences for MIP-1α and MIP-1β in endometriosis [23], suggesting that stimulus-dependent secretory pathways may be required to reveal latent TGF-β1 alterations. The reliance on unstimulated conditions may be particularly limiting for TGF-β1, whose secretion is known to be strongly upregulated by T cell receptor engagement, macrophage activation, and NK cell co-stimulation—all processes absent in a 24 h basal culture [9,17,19]. Similarly, the absence of a TGF-β3 stage gradient may reflect this isoform’s predominantly paracrine and autocrine function within the endometrial stromal compartment rather than systemic immune regulation, with its elevation in the ectopic context being driven by local ESC–peritoneal mesothelium cross-talk not recapitulated in isolated PBMC cultures [1,8]. Thus, the negative TGF-β1 and TGF-β3 findings in our study should be interpreted as reflecting baseline secretory tone rather than total secretory capacity, and should be followed up with stimulus-augmented protocols.

From a biomarker perspective, the present findings have important translational implications. The absence of significant group-level differences for any individual TGF-β isoform in unstimulated PBMC cultures limits the potential of this analyte as a standalone diagnostic marker for endometriosis. CA-125 is the most extensively studied serum biomarker in endometriosis, but its diagnostic performance is insufficient for reliable use across disease stages, exhibits well-documented limitations in sensitivity (approximately 28% for minimal-to-mild disease) and specificity (false positives in adenomyosis, pelvic inflammatory disease, and malignancy) [2,3,23]. Emerging candidates under investigation include BDNF, microRNA profiles, and fucosyltransferase FUT4 expression, but none have achieved validated clinical adoption. Our results suggest that TGF-β2 measured in PBMC supernatants does not meet the threshold for standalone diagnostic utility, but holds promise as a component of a multi-analyte panel for disease severity stratification.

Recent evidence indicates that combined multi-analyte profiling of cytokine species in blood-derived matrices substantially enhances discriminatory power compared with single-analyte approaches [32,33]. Integrating PBMC-derived TGF-β2 with other validated cytokines—such as MIP-1α/β [23], VEGF, FGF-2, PDGF, and proteomic candidates including TGFBI (TGF-β-induced protein ig-h3) and COMP (cartilage oligomeric matrix protein)—within a multiplexed panel may yield the sensitivity and specificity needed for minimally invasive endometriosis diagnosis and monitoring. The recently characterized biomarker TGFBI has demonstrated potential as a plasma-based non-invasive marker for early-stage disease (rASRM I–II), with an area under the curve (AUC) of 0.74 in initial discovery cohorts [9,33]. Adding a PBMC-functional dimension (TGF-β2 basal secretory capacity) to such panels may improve discrimination at advanced stages, where TGFBI alone may be insufficient.

An additional finding warranting mechanistic interpretation is the observed dissociation of TGF-β2 from the TGF-β1/TGF-β3 correlation cluster in the endometriosis group. In controls, all three isoforms were tightly co-regulated (ρ range 0.71–0.78), consistent with their shared transcriptional regulation through SMAD-dependent feedback loops and their common secretion by monocytes and T regulatory cells under homeostatic conditions [8,10,13]. In contrast, within the endometriosis group, the TGF-β1–TGF-β2 correlation was markedly attenuated (ρ = 0.31), whereas TGF-β1–TGF-β3 (ρ = 0.62) and TGF-β2–TGF-β3 (ρ = 0.44) remained moderate. This differential decoupling may reflect partial divergence in the regulation of TGF-β2 relative to TGF-β1 and TGF-β3 in endometriosis.

Mechanistically, TGF-β2 has been shown to be preferentially upregulated by hypoxia-inducible factor 1α (HIF-1α), reactive oxygen species (ROS), and mechanical ECM strain—all conditions prevalent in progressive endometriosis with extensive fibrosis and adhesions [8,12,13]. By contrast, TGF-β1 and TGF-β3 are more strongly regulated by inflammatory cytokines (IL-1β, TNF-α) and T cell activation signals, which may operate independently of fibrosis-driving mechanical stimuli. The observed regulatory dissociation supports the hypothesis that TGF-β2 occupies a distinct, stage-linked niche in the TGF-β isoform network of endometriosis, and that its selective quantification rather than pooled TGF-β measurement may be essential for capturing the fibrotic dimension of disease biology in peripheral blood matrices.

An additional clinically relevant question raised by the significantly higher nulligravidity rate in the endometriosis group (60.0% vs. 36.7%, *p* = 0.04) is whether PBMC-derived TGF-β isoform secretion differs between women with endometriosis who are nulligravid—used here as a proxy for subfertility—and those with prior pregnancies. TGF-β isoforms, particularly TGF-β2 and TGF-β3, have been implicated in endometrial receptivity dysregulation and recurrent implantation failure [34], and TGF-β3, in combination with pro-inflammatory signals, can promote Th17 differentiation at the expense of tolerogenic Treg responses that are critical for embryo implantation [35]. The present study was not designed or powered to address this subgroup question; nulligravidity is furthermore an imprecise surrogate for infertility diagnosis that does not distinguish voluntary nulligravidity from established subfertility or recurrent implantation failure. A dedicated adequately powered analysis prospectively stratifying women by formal infertility diagnosis—ideally distinguishing peritoneal-factor infertility from unexplained infertility—would be needed to evaluate whether TGF-β isoform secretion by PBMCs contributes to the immune dysregulation underlying endometriosis-associated reproductive failure.

The strengths of this study include its prospective design, the use of surgically and histopathologically confirmed endometriosis with detailed rASRM staging, the application of validated multiplex immunoassay technology enabling simultaneous quantification of all three TGF-β isoforms from a single supernatant aliquot, and the systematic exploration of stage-related and inter-isoform patterns in a single analytical framework. The use of unstimulated PBMC cultures provides a reproducible and physiologically clean measure of constitutive secretory phenotype, removing confounders introduced by variable exogenous stimulation conditions across laboratories.

Several limitations must be acknowledged. First, the pilot sample size (n = 50 endometriosis; n = 30 controls), although adequate for primary between-group comparison, limits statistical power for subgroup analyses across individual rASRM stages and increases the risk of type II error in stage-stratified comparisons. Post hoc power analysis for the primary stage-related comparison (TGF-β2, stages I–II vs. III–IV) yielded an observed Cohen’s d of 1.11 (large effect), with an achieved power of 96.9% (α = 0.05), confirming adequate statistical power for this specific comparison despite the pilot sample size. For a confirmatory study, assuming a conservative effect size of d = 0.80 (to account for potential regression to the mean), a minimum of 34 participants per rASRM stage (136 endometriosis cases with balanced stage representation and 136 controls; total N = 272) would be required to achieve 90% power at α = 0.05 (two-tailed). Second, the use of unstimulated cultures may underestimate the TGF-β secretory potential that would be revealed under physiologically relevant stimulation conditions such as phytohemagglutinin (PHA), lipopolysaccharide (LPS), or co-culture with autologous peritoneal fluid. Third, PBMC preparations represent a heterogeneous mixture of T cells, B cells, NK cells, and monocytes, and cell subset-specific contributions to TGF-β isoform secretion cannot be delineated without cell sorting or single-cell approaches. Fourth, while blood collection was preferentially scheduled in the early-to-mid follicular phase (cycle days 5–12) for participants with regular cycles, complete standardization across all participants was not achievable: women with irregular cycles or those for whom scheduling was clinically constrained may have been sampled outside this window. Given that TGF-β2 undergoes an approximately 5-fold increase in endometrial expression during the mid-late secretory phase relative to the follicular phase, residual cycle-phase variability in a subset of participants may have introduced additional noise into the TGF-β2 stage-related analysis. Sensitivity analyses stratifying by cycle phase were not pre-planned and are precluded by the absence of systematic LMP documentation for all participants; confirmatory studies should prospectively verify follicular-phase sampling by serum estradiol and progesterone measurement at the time of blood collection. Fifth, peritoneal fluid was not collected in parallel, precluding direct compartmental comparison of TGF-β levels between the systemic and local environment in the same patient. Sixth, PBMC cultures were performed in RPMI-1640 supplemented with 10% heat-inactivated fetal bovine serum (FBS), which contains approximately 1000–2000 pg/mL of latent TGF-β at working concentration. Because the Bio-Plex protocol requires sample acidification to detect both active and latent TGF-β forms, FBS-derived latent TGF-β is co-detected alongside PBMC-secreted cytokine, meaning that the absolute concentrations reported here cannot be attributed exclusively to PBMC secretion. Critically, however, all cultures were performed using medium supplemented with FBS from the same lot and manufacturer (Sigma-Aldrich, St. Louis, MO, USA), creating a fixed and symmetric background signal across all participants and both study groups. Between-group comparisons (endometriosis vs. controls) and stage-stratified comparisons (rASRM stages I–II vs. III–IV) therefore remain internally valid, since the FBS-derived background cancels symmetrically and cannot generate or mask a differential signal between groups.

Furthermore, although controls were recruited exclusively from women undergoing laparoscopy with no macroscopic evidence of endometriosis—representing the most accurate available method for disease exclusion—a theoretical residual risk of misclassification cannot be entirely excluded, as histopathological confirmation of the entire peritoneal surface was not performed in all control participants. This potential source of non-differential misclassification would, if present, bias between-group TGF-β comparisons toward the null and could contribute to the absence of significant group-level differences observed in the present study.

### Future Directions

Future studies should address these limitations through several complementary strategies. First, stimulus-augmented culture protocols—employing PHA, recombinant IL-2, or autologous peritoneal fluid as conditioning stimuli—should be applied to determine whether latent TGF-β alterations become manifest upon immune activation. Second, cell subset-resolved analyses using fluorescence-activated cell sorting (FACS) or single-cell RNA sequencing of PBMCs would clarify which immune cell populations contribute to the stage-dependent TGF-β2 signal. Third, parallel collection of peritoneal fluid and peripheral blood from the same patients would enable direct quantification of the compartmentalization gradient and identification of patients with peritoneal-to-systemic TGF-β overflow. Fourth, integration of TGF-β isoform data with multi-omics panels—including proteomic, miRNA, and metabolomic profiling—may yield composite biomarker signatures with sufficient diagnostic accuracy for clinical translation [9,33]. Fifth, longitudinal sampling designs would determine whether PBMC TGF-β2 levels change dynamically with disease progression or therapeutic response, supporting its potential utility as a monitoring biomarker in endometriosis management. Sample size calculations for confirmatory studies should target a minimum of 150 cases with balanced stage representation and cycle-phase standardization at collection. Sixth, future protocols should transition from FBS-supplemented RPMI to serum-free or TGF-β-depleted FBS media to eliminate bovine TGF-β isoform contribution to the measured supernatant signal, and parallel measurement of TGF-β concentrations in cell-free medium blanks at t = 0 and t = 24 h should be incorporated as a standard quality-control procedure enabling precise subtraction of the serum-derived background.

Technical recommendations for future studies. Future studies should transition from standard FBS-supplemented RPMI to reduced-serum (1–2% FBS) or serum-free culture media (e.g., X-VIVO 15, CTS OpTmizer), or employ TGF-β-depleted FBS prepared via anti-LAP immunodepletion, to enable unambiguous attribution of supernatant TGF-β signals to PBMC secretion. Parallel measurement of TGF-β concentrations in cell-free medium blanks at baseline (t = 0) and at 24 h (t = 24 h) should be incorporated as a standard quality-control procedure enabling precise subtraction of the serum-derived background from each experimental supernatant [36].

## 4. Materials and Methods

### 4.1. Study Design and Ethical Approval

This prospective pilot case–control study was conducted at a single tertiary referral center (Department of Gynecology, Obstetrics and Gynecologic Oncology, Medical University of Silesia, Katowice, Poland) between 2017 and 2020. The study was conducted in accordance with the Declaration of Helsinki and approved by the Bioethics Committee of the Medical University of Silesia, Katowice, Poland (protocol code KNW/0022/KB/110/0/17, approved 3 October 2017). Written informed consent was obtained from all participants prior to inclusion.

### 4.2. Participants

Inclusion criteria for the endometriosis group were: (i) premenopausal women aged 18–50 years; (ii) laparoscopic diagnosis of endometriosis with intraoperative rASRM staging; and (iii) histopathological confirmation of excised lesions. Exclusion criteria included: autoimmune disease, active infection, or inflammatory condition, immunosuppressive or hormonal therapy within 3 months of sampling, current pregnancy, and systemic malignancy. For participants, blood collection and laparoscopy were preferentially scheduled between cycle days 5 and 12 (counted from the first day of the last menstrual period), thereby sampling participants predominantly in the early-to-mid follicular phase and minimizing intra-group variability attributable to cyclical TGF-β fluctuation. Controls were premenopausal women without clinical or laparoscopic evidence of endometriosis, pelvic inflammatory disease, or autoimmune conditions, recruited from women undergoing laparoscopy for benign gynecological indications (e.g., ligation, diagnostic evaluation). All participants fasted for a minimum of 8 h before blood collection.

### 4.3. Blood Collection and PBMC Isolation

Peripheral venous blood (20 mL) was collected by standardized venipuncture immediately prior to laparoscopy, before any anesthetic agents were administered. PBMCs were isolated by density gradient centrifugation using Ficoll-Paque PLUS (GE Healthcare, Chicago, IL, USA) according to the manufacturer’s protocol. Briefly, blood was diluted 1:1 with phosphate-buffered saline (PBS), layered over Ficoll-Paque, and centrifuged at 400× *g* for 30 min at room temperature. The buffy coat layer was aspirated, washed twice with PBS, and resuspended in complete RPMI-1640 medium (supplemented with 10% heat-inactivated fetal bovine serum, 2 mM L-glutamine, 100 U/mL penicillin, and 100 μg/mL streptomycin; all from Sigma-Aldrich, St. Louis, MO, USA). Cell viability was assessed by trypan blue exclusion and was ≥95% in all preparations.

### 4.4. In Vitro PBMC Culture

Cells were seeded at 2 × 10^6^ cells/mL in 4-well plates (1 mL/well) and incubated for 24 h at 37 °C in a humidified atmosphere with 5% CO_2_ without exogenous stimulation. Cultures were performed in duplicate for each participant. After 24 h, supernatants were collected by centrifugation at 300× *g* for 10 min, aliquoted, and stored at −80 °C until analysis. Freeze–thaw cycles were limited to one per aliquot. It is acknowledged that the use of 10% FBS-supplemented medium introduces a background of latent TGF-β that is co-detected following the acidification step required by the Bio-Plex protocol; this methodological limitation is addressed in detail in the Limitations section (Section 3, paragraph 6).

### 4.5. Cytokine Quantification

Concentrations of TGF-β1, TGF-β2, and TGF-β3 in culture supernatants were measured simultaneously using a multiplex bead-based immunoassay (Bio-Plex Pro TGF-β Panel; Bio-Rad Laboratories, Hercules, CA, USA) based on xMAP Luminex technology, according to the manufacturer’s protocol. Samples were acidified prior to assay to detect both active and latent TGF-β forms, as recommended by the manufacturer. Each sample was assayed in duplicate; the mean of the duplicate measurements was used for analysis. Standard curves were generated for each analyte using recombinant TGF-β standards supplied with the Bio-Plex Pro TGF-β Panel kit by the manufacturer (Bio-Rad Laboratories, Hercules, CA, USA), and concentrations were calculated by 5-parameter logistic (5PL) regression. Results are expressed as pg/mL.

### 4.6. Statistical Analysis

Data were tested for normality using the Shapiro–Wilk test. As concentrations were not normally distributed, non-parametric methods were applied throughout. Between-group comparisons (endometriosis vs. controls) for each isoform were performed using the Mann–Whitney U test. Differences across rASRM stages within the endometriosis cohort were assessed using the Kruskal–Wallis test, followed by Dunn’s post hoc test with Bonferroni correction for multiple comparisons. Associations between TGF-β isoform concentrations and continuous clinical variables (age, gravidity, parity) were evaluated using Spearman’s rank correlation coefficient (ρ). Inter-isoform correlations were computed separately within each group. All tests were two-tailed; statistical significance was defined as α = 0.05. Statistical analyses were performed using Statistica v.13 (TIBCO Software Inc., Palo Alto, CA, USA) and GraphPad Prism v.9 (GraphPad Software, San Diego, CA, USA).

## 5. Conclusions

Unstimulated in vitro TGF-β secretion by circulating PBMCs is not globally elevated in women with endometriosis compared with disease-free controls, suggesting that TGF-β dysregulation in this condition is primarily compartmentalized to the peritoneal microenvironment rather than systemically imprinted in the peripheral immune compartment. This finding supports the view that local peritoneal stimuli play a major role in driving the immunosuppressive and fibrogenic TGF-β output characteristic of endometriosis pathophysiology.

Despite the absence of overall group differences, TGF-β2 secretion by PBMCs exhibited a stage-dependent increase in women with moderate-to-advanced disease (rASRM stages III–IV) compared with minimal-to-mild disease (stages I–II), and its correlation with TGF-β1 and TGF-β3 was attenuated in the endometriosis group— a pattern compatible with selective isoform-specific regulation in advanced disease, but requiring mechanistic confirmation. These results support a putative role for PBMC-derived TGF-β2 as a candidate biomarker of disease severity and fibrotic burden, rather than a diagnostic marker for primary endometriosis detection.

Larger prospective studies integrating stimulus-augmented PBMC culture protocols, parallel peritoneal fluid sampling, cell subset-resolved cytokine profiling, and multi-analyte panel approaches are warranted to validate the clinical utility of TGF-β2 in the non-invasive monitoring of endometriosis and to fully characterize the peripheral immune secretory landscape of this complex disease.

## Figures and Tables

**Table 1:** Baseline clinical and demographic characteristics of study participants.

Characteristic	Endometriosis (n = 50)	Control (n = 30)	*p*-Value
Age, years (mean ± SD)	34.2 ± 8.2	37.5 ± 11.7	0.45 (NS)
Age, years (median)	35.0	35.5	
Nulligravid, n (%)	30 (60.0%)	11 (36.7%)	0.04
rASRM stage I, n (%)	12 (24%)	—	—
rASRM stage II, n (%)	14 (28%)	—	—
rASRM stage III, n (%)	13 (26%)	—	—
rASRM stage IV, n (%)	11 (22%)	—	—

NS = not significant; rASRM = revised American Society for Reproductive Medicine; SD = standard deviation; — = not applicable.

**Table 2:** TGF-β isoform concentrations in 24 h unstimulated PBMC culture supernatants.

Cytokine	Endometriosis (Median, IQR) pg/mL	Control (Median, IQR) pg/mL	*p*-Value
TGF-β1	103,816 (90,955–125,630)	114,700 (96,800–121,671)	0.25
TGF-β2	3735 (3142–4204)	3732 (3362–4179)	0.32
TGF-β3	3280 (3027–3463)	3284 (3050–3477)	0.70

IQR = interquartile range; between-group comparisons by Mann–Whitney U test; *p*-values are two-tailed.

**Table 3:** Stage-related TGF-β isoform concentrations within the endometriosis cohort.

Cytokine	Stage I (n = 12) Median (IQR)	Stage II (n = 14) Median (IQR)	Stage III (n = 13) Median (IQR)	Stage IV (n = 11) Median (IQR)
TGF-β1 (pg/mL)	101,204 (88,250–119,600)	104,516 (91,305–122,800)	105,820 (93,100–128,340)	102,980 (89,500–124,100)
TGF-β2 (pg/mL)	3342 (3012–3680) *	3590 (3210–3920) *	3980 (3540–4350) †	4210 (3780–4650) †
TGF-β3 (pg/mL)	3195 (3001–3380)	3260 (3050–3490)	3310 (3070–3510)	3340 (3100–3520)

Values are median (interquartile range). * = Stages I–II (minimal/mild group); † = Stages III–IV (moderate/advanced group); significant difference between minimal/mild vs. moderate/advanced for TGF-β2 only (post hoc Dunn test, *p* < 0.05). All TGF-β1 and TGF-β3 comparisons: *p* > 0.05.

## Data Availability

The original contributions presented in this study are included in the article. Further inquiries can be directed to the corresponding author.

## Abbreviations

AUC	area under the curve
BDNF	brain-derived neurotrophic factor
CA-125	cancer antigen 125
COMP	cartilage oligomeric matrix protein
DIE	deep infiltrating endometriosis
ECM	extracellular matrix
EMT	epithelial-to-mesenchymal transition
ESC	endometrial stromal cell
FACS	fluorescence-activated cell sorting
FAK	focal adhesion kinase
HIF-1α	hypoxia-inducible factor 1 alpha
ILK	integrin-linked kinase
IQR	interquartile range
MMP	matrix metalloproteinase
NK	natural killer
OMA	ovarian endometrioma
PBMC	peripheral blood mononuclear cell
rASRM	revised American Society for Reproductive Medicine
RIF	repeated implantation failure
ROS	reactive oxygen species
SD	standard deviation
SMAD	small mothers against decapentaplegic
TGF-β	transforming growth factor-beta
TGFBI	TGF-β-induced protein ig-h3
TGFBR1	TGF-β receptor type 1 (ALK5)
TGFBR2	TGF-β receptor type 2
Treg	regulatory T cell
TVS	transvaginal ultrasound
VEGF	vascular endothelial growth factor
5PL	five-parameter logistic
